# Supplementary material for: Non-propagating RNA virus-vectored HA/NA vaccine prevents shedding of antigen-drifted H1N1 influenza virus in pigs
Source: NPJ Vaccines. 2026 Mar 28;11:104. doi: 10.1038/s41541-026-01428-6 (PMC13194767; doi:10.1038/s41541-026-01428-6)
Supplement: Supplementary file 1 — Supplementary information [file 41541_2026_1428_MOESM1_ESM.pdf]

Non-propagating RNA virus-vectored HA/NA vaccine prevents shedding of antigen-drifted H1N1 influenza virus in pigs

Obdulio Garcia-Nicolas[1,2], Lisa Butticaz[1], Robin Avanthay[1], Nicolas Ruggli [1,2], Artur Summerfield[1,2], Gert Zimmer[1,2]

[1] Institute of Virology and Immunology IVI, Mittelhäusern, Switzerland,

[2] Department of Infectious Diseases and Pathobiology, Vetsuisse Faculty, University of Bern, Bern, Switzerland,

Correspondence to: Dr. Gert Zimmer (gert.zimmer@ivi.admin.ch)

Supplementary Information

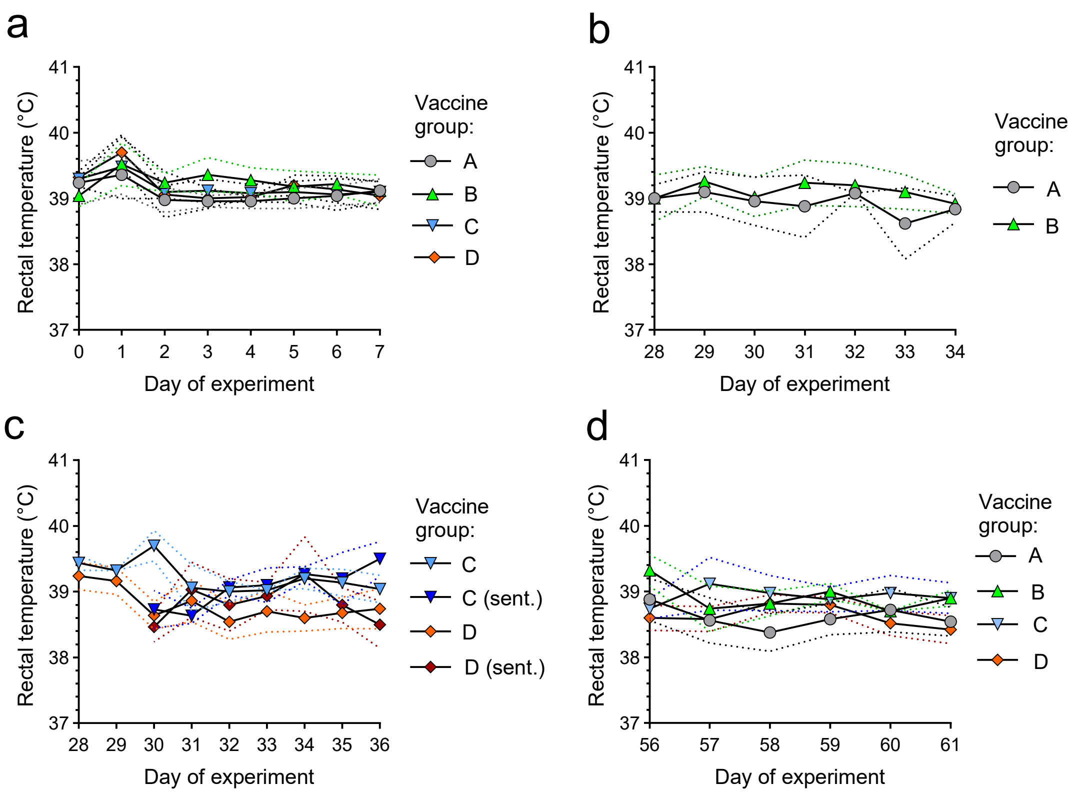

Supplementary Fig. 1 | Recording of the rectal temperature in SPF pigs following vaccination and challenge infection. **a-d** The rectal temperature was recorded in all animals of the indicated groups (n = 5 animals/group) on the indicated day of the experiment. Mean values with SD (dotted lines) are shown. **a** Recording of rectal temperature following prime vaccination of groups A - D. **b** Recording of the rectal temperature in animals of groups A and B following boost vaccination. **c** Recording of rectal temperature in animals of group C and D (n = 5/group) after receiving the LAIV and in the sentinel pigs (n = 3) that were co-housed with each of the groups. **d** Recording rectal temperature in pigs of all groups following nasal challenge infection with the $H1N1_{HH4/09}$/$H1N1_{Vic/19}$ 6:2 reassortant virus.

HA H1N1$_{HH4/09}$ 1 MKAILVV**L**LYTFTTANADTLCIGYHANNSTDTVDTVLEKNVTVTHSVNLLEDKHNGKLCKLRGVAPLHLGKCNIAGWILG

HA H1N1$_{Vic/19}$ 1 ....**M**..

HA H1N1$_{HH4/09}$ 81 NPECESLSTA**S**SWSYIVETS**S**SDNGTCYPGDFI**D**YEELREQLSSVSSFERFEIFPKTSSWPNHDS**NK**GVTAACPHAGAKS

HA H1N1$_{Vic/19}$ 81 ..........**R**.........**N**............**N**...............................**DN**.............

HA H1N1$_{HH4/09}$ 161 FYKNLIWLVKKG**N**SYPK**LSKS**YINDKGKEVLVLWGIHHP**S**T**S**ADQQSLYQNADAYVFVG**S**SRYSKKFKPEIA**I**RPKVRD**Q**

HA H1N1$_{VIC/19}$ 161 ............**K**....**INQT**..................**P**.**I**.................**T**............**T**......**R**

HA H1N1$_{HH4/09}$ 241 EGRMNYYWTLVEPGDKITFEATGNLV**V**PRYAF**A**MER**N**AGSGIIISDTPVHDCNTTCQTP**K**GAINTSLPFQN**I**HPITIGKC

HA H1N1$_{VIC/19}$ 241 ..........................**A**.....**T**...**D**......................**E**...........**V**........

HA H1N1$_{HH4/09}$ 321 PKYVKSTKLRLATGLRNVPSIQSRGLFGAIAGFIEGGWTGMVDGWYGYHHQNEQGSGYAADLKSTQNAID**E**ITNKVNSVI

HA H1N1$_{VIC/19}$ 321 ....**K**.........

HA H1N1$_{HH4/09}$ 401 EKMNTQFTAVGKEFNHLEKRIENLNKKVDDGFLDIWTYNAELLVLLENERTLDYHDSNVKNLYEKVR**S**QLKNNAKEIGNG

HA H1N1$_{VIC/19}$ 401 ....**N**............

HA H1N1$_{HH4/09}$ 481 CFEFYHKCDNTCMESVKNGTYDYPKYSEEAKLNRE**E**IDGVKL**E**STRIYQILAIYSTVASSLVLVVSLGAISFWMCSNGSL

HA H1N1$_{VIC/19}$ 481 ...................................**K**......**D**.....................................

HA H1N1$_{HH4/09}$ 561 QCRICI

HA H1N1$_{VIC/19}$ 561 ......

Supplementary Fig. 2 | Alignment of HA primary amino acid sequences. The HA sequences of A/Hamburg/4/2009 (H1N1) (GenBank accession no. ACR10223) and A/Victoria/2570/2019 (H1N1) (GenBank accession no. WEY08940) are shown. The predicted signal sequence (amino acids 1-17) and transmembrane domain (amino acids 537-560) are depicted with a grey background. Amino acid positions differing between the two HA antigens are shown in bold letters. The amino acid identity of the two HA antigens is 95.5%.

```
NA H1N1_HH4/09    1 MNPNQKIITIGSVCMTIGMANLILQIGNIISTWISHSIQLGNQNQIETCNQSVITYENNTWVNQTYVNISNTNFAAGQSV
NA H1N1_VIC/19    1 ............I.....T............I.V.....I...S......K..............F.......S..R...
NA H5N1_TEX/24    1 .......T....I..V..IVS.M........I.V.....T...Y.P.P....I.............I.......L.E.A.
NA H1N1_HH4/09   81 VSVKLAGNSSLCPVSGWAIYSKDNSVRIGSKGDVFVIREPFISCSPLECRTFFLTQGALLNDKHSNGTIKDRSPYRTLMS
NA H1N1_VIC/19   81 A...............................................................................
NA H5N1_TEX/24   81 T..T.........I..........GI...................H......................V...........
NA H1N1_HH4/09  161 CPIGEVPSPYNSRFESVAWSASACHDGINWLTIGISGPDNGAVAVLKYNGIITDTIKSWRNNILRTQESECACVNGSCFT
NA H1N1_VIC/19  161 ...........................T...........S.....................K..................
NA H5N1_TEX/24  161 ..V..A......................S...................................................
NA H1N1_HH4/09  241 VMTDGPSNGQASYKIFRIEKGKIVKSVEMNAPNYHYEECSCYPDSSEITCVCRDNWHGSNRPWVSFNQNLEYQIGYICSG
NA H1N1_VIC/19  241 I......D...............I.....K...........................................M......
NA H5N1_TEX/24  241 ................K.....V.....................AGD.M...............................
NA H1N1_HH4/09  321 IFGDNPRPNDKTGSCGPVSSNGANGVKGFSFKYGNGVWIGRTKSISSRNGFEMIWDPNGWTGTDNNFSIKQDIVGINEWS
NA H1N1_VIC/19  321 V...............................................K................K..K...........
NA H5N1_TEX/24  321 ..........GT.....MP....Y....................T...S............E..SS..V.....E.TD..
NA H1N1_HH4/09  401 GYSGSFVQHPELTGLDCIRPCFWVELIRGRPKENTIWTSGSSISFCGVNSDTVGWSWPDGAELPFTIDK
NA H1N1_VIC/19  401 ...............N...............E................D..I.................
NA H5N1_TEX/24  401 .................M...................................................
```

Supplementary Fig. 3 | Alignment of NA primary amino acids sequences. Alignment of the NA amino acid sequences of A/Hamburg/4/2009 (H1N1) (GenBank accession no. ACR10227), A/Victoria/2570/2019 (H1N1) (GenBank accession no. WEY08939), and A/cattle/Texas/063224-24-1/2024 (H5N1) (GISAID acc. no. EPI_ISL_19155861) is shown. The predicted transmembrane domains (amino acids 7-30) are depicted with a grey background. Amino acid positions differing between the two NA antigens are shown in bold letters.

Supplementary Table 1 | Clinical score of SPF pigs following boost vaccination.

Animal no.	Clinical score at the indicated day of the experiment [a]								
	**d28**	**d29**	**d30**	**d31**	**d32**	**d33**	**d34**	**d35**	**d36**
**A1**	0	**2**	**1**	0	0	0	0		
**A2**	0	**2**	**1**	0	0	0	0		
**A3**	0	1	**1**	0	0	0	0		
**A4**	0	**2**	**1**	0	0	0	0		
**A5**	0	**2**	**1**	0	0	0	0		
**B1**	0	**2**	**1**	0	0	0	0		
**B2**	0	0	**1**	0	0	0	0		
**B3**	0	0	1	0	0	0	0		
**B4**	0	0	0	0	0	0	0		
**B5**	0	0	0	0	0	0	0		
**C1**	0	0	0	0	**3**	**3**	**1**	0	0
**C2**	0	0	0	0	0	**3**	**1**	0	0
**C3**	0	0	0	0	0	**3**	**1**	0	0
**C4**	0	0	0	0	0	**3**	**1**	0	0
**C5**	0	0	0	**2**	**3**	**3**	**1**	0	0
**SC1** [b]			0	0	**2**	**3**	0	0	0
**SC2**			0	0	0	**3**	**2**	0	0
**SC3**			0	0	**2**	**3**	0	0	0
**D1**	0	0	0	0	0	0	0	0	0
**D2**	0	0	0	0	0	0	0	0	0
**D3**	0	0	0	0	0	0	0	0	0
**D4**	0	0	0	0	0	0	0	0	0
**D5**	0	0	0	0	0	0	0	0	0
**SD1**			0	0	0	**0**	0	0	0
**SD2**			0	0	0	0	0	0	0
**SD3**			0	0	0	0	0	0	0

[a] Clinical symptoms of disease were rated 0 (no symptoms), 1 (mild symptoms), 2 (moderate symptoms), 3 (severe symptoms). The total score corresponds to the cumulative sum of all symptoms rated and was assigned for each animal at each day of the survey.
[b] SC, sentinel pigs co-housed with group C animals.
[c] SD, sentinel pigs co-housed with group D animals.

Supplementary Table 2 | Clinical scoring of SPF pigs following challenge infection with H1N1$_{Vic/19}$.

Animal no.	Clinical score at the indicated day post-challenge [a]					
	**d56**	**d57**	**d58**	**d59**	**d60**	**d61**
**A1**	0	0	**1**	**1**	0	0
**A2**	0	0	**1**	0	0	0
**A3**	0	0	**1**	**1**	0	0
**A4**	0	0	**1**	**1**	**1**	0
**A5**	0	0	**1**	**1**	**1**	0
**B1**	0	0	**1**	0	0	0
**B2**	0	0	**1**	0	0	0
**B3**	0	0	0	0	0	0
**B4**	0	0	**1**	0	0	0
**B5**	0	0	**1**	0	0	0
**C1**	0	0	0	0	0	0
**C2**	0	0	0	0	0	0
**C3**	0	0	0	0	0	0
**C4**	0	0	0	0	0	0
**C5**	0	0	0	0	0	0
**D1**	0	0	0	0	0	0
**D2**	0	0	0	0	0	0
**D3**	0	0	0	0	0	0
**D4**	0	0	0	0	0	0
**D5**	0	0	0	0	0	0

[a] Clinical symptoms of disease were rated 0 (no symptoms), 1 (mild symptoms), 2 (moderate symptoms), 3 (severe symptoms). The total score corresponds to the cumulative sum of all symptoms rated and was assigned for each animal at all days after challenge infection.
